# Association between susceptible genotypes to periodontitis and clinical outcomes of periodontal regenerative therapy: A systematic review

**DOI:** 10.4317/medoral.21105

**Published:** 2016-03-06

**Authors:** Georgios-Sokratis Chatzopoulos, Vasiliki-Petros Koidou

**Affiliations:** 1 DDS, Resident, Advanced education program in Periodontology, University of Minnesota, Minneapolis, Minnesota, USA

## Abstract

**Background:**

The aim of this review is to systematically investigate the effect of a susceptible genotype to periodontitis with the clinical outcomes of periodontal regeneration.

**Material and Methods:**

Based on a focused question, an electronic search identified 155 unique citations. Three journals (Journal of Periodontology, Journal of Clinical Periodontology and Journal of Periodontal Research), references of relevant studies and review articles were hand-searched. Two independent reviewers implementing eligibility inclusion criteria selected the studies.

**Results:**

Of the 155, four studies fulfilled the inclusion criteria. All studies were published between 2000 and 2004 and the samples’ size was 40 to 86 patients. Polymorphisms of Interleukin-1 (IL-1) gene were included in all. Three out of four studies failed to identify an association between susceptible genotypes to periodontitis and clinical outcomes of periodontal regeneration, while one found an association. The heterogeneity and small number of studies included prevented the conduct of a meta-analysis. No studies were identified evaluating the effect of other genotypes and as a result only IL-1 genotype studies were included.

**Conclusions:**

Within the limits of the present review, no direct conclusion for the effect of a susceptible IL-1 genotype status to the clinical outcome after periodontal regeneration could be drawn. The need of more qualitative studies to explore a possible association emerges.

**Key words:**Periodontitis, genotype, periodontal therapy, regeneration, susceptibility, systematic review.

## Introduction

Periodontal therapy aims in reducing the tissue inflammation caused by bacterial plaque, preventing further loss of attachment while aiming as well in regenerating the anatomical bone structures around teeth (1,2). Non-surgical as well as surgical periodontal treatment results in a reduction of periodontal probing depth thus facilitating patient’s oral hygiene (2). However periodontal treatment does not provide or provides minimal regeneration of the compromised tissues. Consequently, other surgical procedures, such as osseous grafting and guided tissue regeneration, emerged as means of providing a more predictable and long term stable result in regeneration of the periodontal tissues (1).

Bone replacement graft, is one of the most commonly encountered therapeutic approaches when dealing with osseous defects (3). Autogenous bone grafts, from extra-oral or intra-oral donor sites, allogenic bone grafts, alloplasts and xenografts are all used in periodontal osseous defects (1). Bone grafting is considered a better therapeutic technique for intrabony defects than open flap debridement, while when combined with the use of membranes even better clinical outcomes have been reported (3). The clinical outcome of bone replacement graft may vary. The type of graft biomaterials and the techniques used, such as the flap design, suturing technique and the anatomy of the defect, affect the outcome (4).

Guided tissue regeneration (GTR) is a periodontal surgical procedure introduced in the early 80s aiming in the regeneration of all the anatomical structures around the teeth, including the cementum, periodontal ligament and alveolar bone (1,5). In GTR, the use of a biocompatible barrier membrane (resorbable or nonresorbable) assures the maintenance of an adequate space for bone and other attachment tissues to cover the osseous defect and prevent the migration of the epithelial and gingival connective tissue (2). GTR is reported to result in a greater gain of clinical attachment, probing depth reduction and filling of deep intrabony defects when compared to open flap debridement as presented in two systematic reviews (6,7). The clinical outcome of periodontal regeneration with GTR may be affected by smoking, high plaque scores, bleeding on probing, deep periodontal pocket depth and the anatomical characteristics of the defect (2).

Enamel Matrix proteins were also found to regenerate the periodontal tissues since amelogenins were found to mimic the events during the development of the periodontal tissues (8,9). The cells of the Hertwig’s epithelial root sheath were found to produce enamel matrix proteins on the root surface before the cementum formation (9). Enamel matrix derivative (EMD) was found to provide an additional gain of clinical attachment levels of 1.3mm as well as provide greater reduction of probing depth that ranged from 1.0 mm to 1.6 mm (4). The use of EMD is presented as equally effective as guided tissue regeneration (4).

Back in 1966 a study counting over 1800 individuals, identified the presence of a subgroup of patients who possessed a greater risk of developing periodontitis (10). Later, Michalowicz *et al.* (11) found that a variance of periodontal status could be attributed to genetic factors in 38% to 82% of the population. An increasing number of studies strongly associate some host genetic factors with the onset of periodontal disease (12). Interleukin-1 (IL-1) is one of the most studied polymorphism of the human genome that influences periodontal disease. The influence of susceptible genotypes of IL-1 in periodontal therapy outcome and progression has recently drawn attention (13,14).

Similarly, a susceptible genotype to periodontal disease may influence the outcome of the periodontal regeneration as well. Hence, the purpose of this study was to assess systematically the association between susceptible genotypes to periodontitis and the clinical outcomes of periodontal regeneration therapy.

## Material and Methods

- Review question

What is the effect of a susceptible genotype to periodontitis following periodontal regeneration therapy on the clinical parameters of periodontal disease, such as probing depth (PD), clinical attachment level (CAL), gingival recession (REC), bleeding on probing (BOP) and plaque index (PI), when compared to non-susceptible genotypes?

- Literature review

A search was undertaken to identify all clinical studies that investigated the association between the susceptibility to periodontitis genotypes and the clinical outcomes of periodontal regeneration treatment.

Longitudinal clinical studies that were published up to May 2014 (week 4) were identified electronically (MEDLINE, SCOPUS Elsevier and Cochrane Library). The search was conducted in May (week 4) using ten (10) keywords and included papers published up until that time point. The terms “Periodontal regeneration”, “Regenerative periodontal therapy”, “Guided tissue regeneration”, “Guided bone regeneration”, “Periodontal therapy”, each joined by the connector OR were linked by the Boolean connector AND to the terms “polymorphism, genetic”, “polymorphism”, “genotype”, “haplotype”. In three journals (3) (Journal of Periodontology, Journal of Clinical Periodontology and Journal of Periodontal Research), references of all relevant studies and review articles were hand-searched. No specific language restriction was applied to any of the searches.

- Study selection

Two investigators (G.C,V.K) screened independently the titles and abstracts of all the articles identified in the electronic and manual search. Articles which met the following inclusion criteria were included in the review:

● Prospective and retrospective cohort studies.

Including systemically healthy patients, aged >18 years and with at least one infrabony defect.

● Studies relating the influence of susceptible genotypes to periodontitis on the clinical outcome of periodontal regenerative therapy.

● An observation time period of at least 6 months after therapy was mandatory.

● Evaluation of the clinical outcome with the use of one or more of the clinical parameters: probing pocket depth (PPD), clinical attachment level (CAL), bleeding on probing scores (BOP), plaque index (PI), gingival recession (REC) before and after therapeutic procedure;

No language restriction was applied. However, electronic search included at least a title in English. None of the articles which met the inclusion criteria was written in a language other than English. In case of disagreement between the reviewers, discussion was utilised for resolution.

- Data extraction

The data was extracted independently by the two reviewers (G.C and V.K) and was presented into two tables consisting of general information for the studies (author, year of publication, journal), source of the sample, location of the polymorphism, studies’ protocols, clinical parameters that were used to evaluate the periodontal regeneration therapy outcomes and the studies’ conclusions. A second table was used to extract data related to the sample’s characteristics depending on the genotype profile (positive or negative), such as age, distribution of the sample with respect to gender, systemic condition, administration of antibiotics, smoking habits, regeneration site, defect morphology and ethnicity. In the same table the duration of the follow-up was recorded.

The quality assessment of the included case control studies was assessed with the use of the Newcastle-Ottawa Scale (NOS) (15). The modified NOS was used as described by Chambrone *et al.* (16) and assessment of selection, comparability, exposure and statistical bias were performed.

## Results

**Figure 1:**
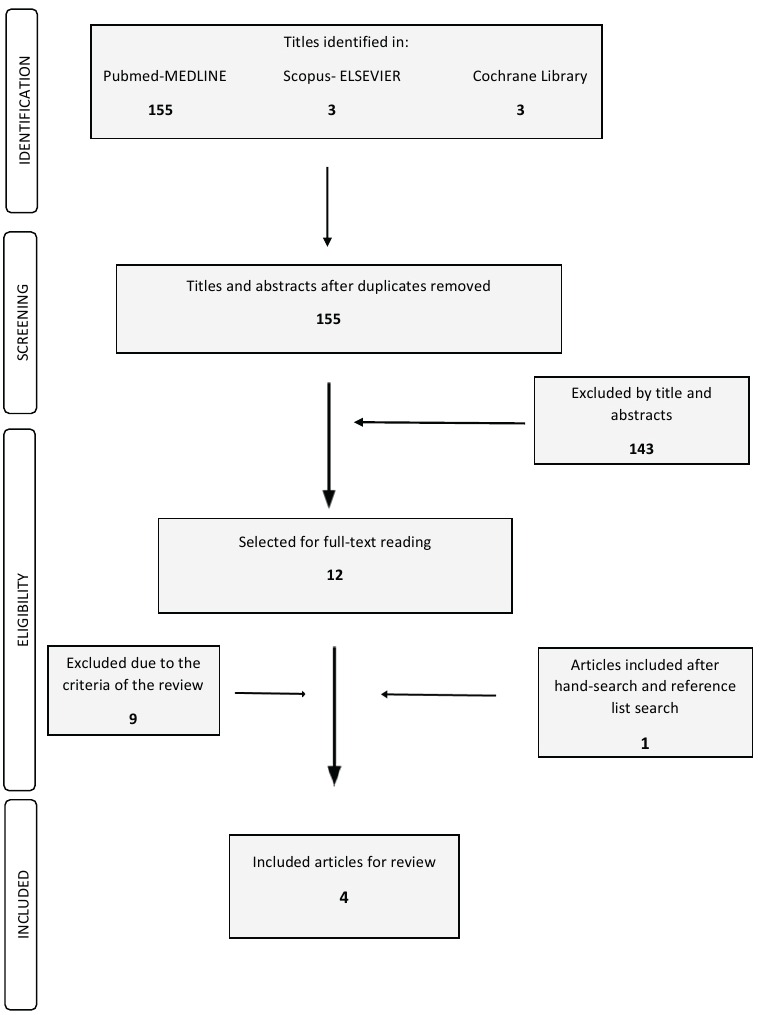
Search and selection results.

Initial electronic searches identified 155 publications. After screening of the titles and abstracts independently, 12 (14,17-27) studies were selected for full-text review (Fig. 1). The other 143 publications were excluded due to the irrelevant topic. All identified papers in first selection included at least a title in English. None of the included papers were in a language other than English and thus translation of the abstract or/and the main text was not required for any paper. Inter reviewer agreement at the first phase of selection was very high (k = 0.92). After full-text and citation mining, four articles were identified as determined by the inclusion and exclusion criteria (k=0.98). Of the four articles, three (25-27) were initially identified by electronic search, while the other one (28) by citation mining.

The review of the 12 full text studies resulted in the exclusion of nine articles (14,17-24) due to lack of periodontal regeneration intervention included in the study protocol. All of those studies employed open flap debridement without periodontal regeneration. At the same time, only studies evaluated the influence of the presence of IL-1 positive genotype on the clinical outcome of periodontal regenerative therapy were identified in the literature.

- Study characteristics are presented in  Table 1, Table 2.

**Table 1:**
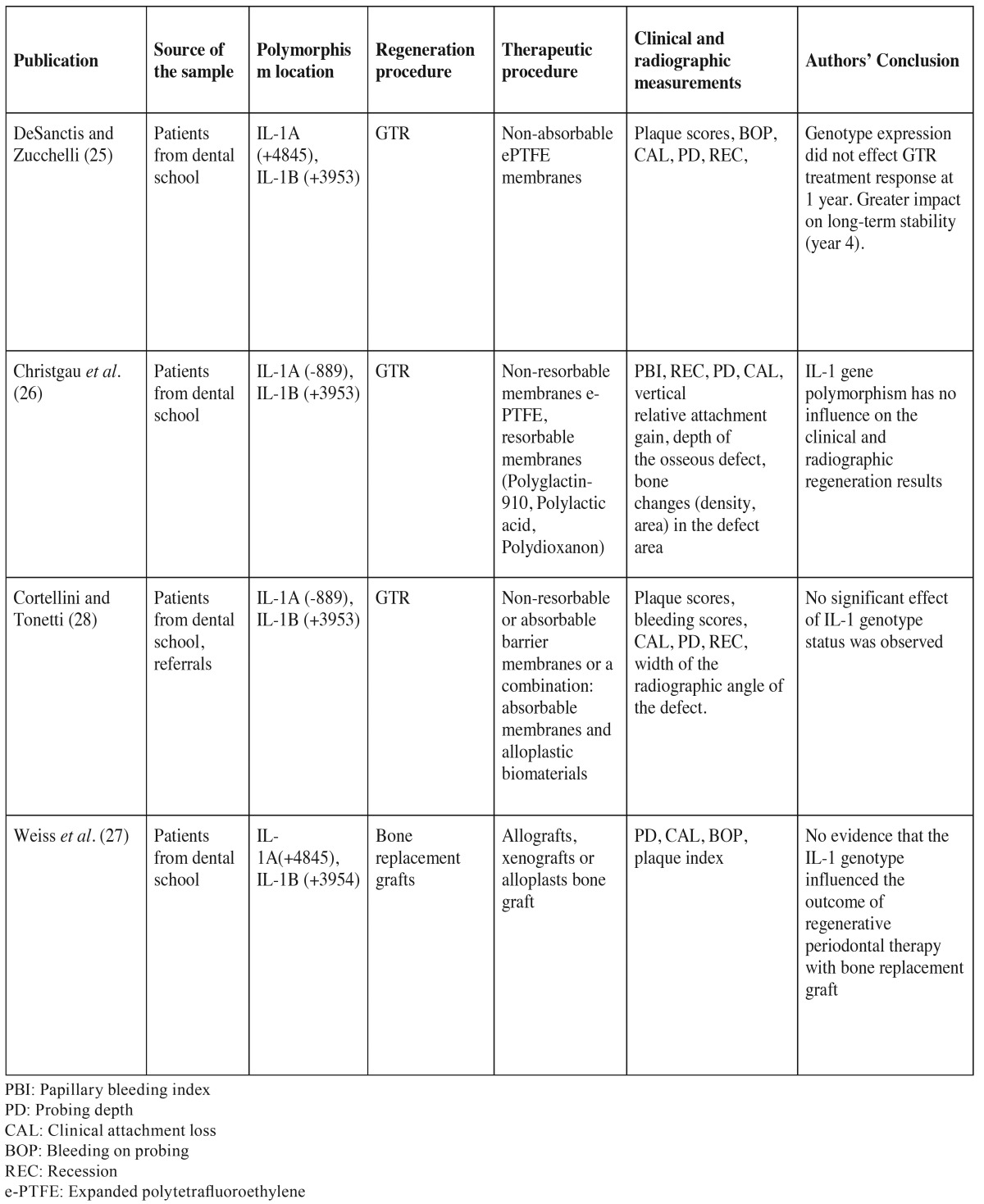
Characteristics of the publications.

**Table 2:**
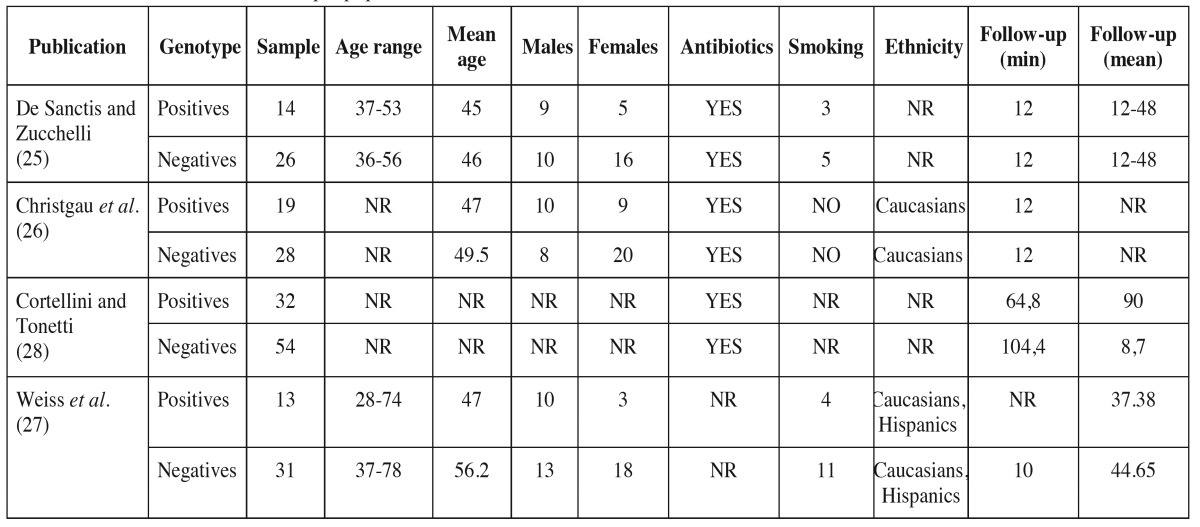
Characteristics of the sample population.

The studies were published between 2000 and 2003 and the included sample was patients from dental schools. The effect of IL-1 genotype status on the clinical outcome of the guided tissue regeneration (25,26,28) and the bone replacement grafts (27) was investigated in the studies. One study was prospective cohort (25), while the other three were retrospective cohort studies.

The first study was published by DeSanctis and Zucchelli in 2000 (25), and included 14 patients with positive genotype to periodontitis and a mean age of 45 years. All of them were systematically healthy and three were smokers. The minimum follow-up was 12 months and ranged between 12 and 48 months. The investigators used non-absorbable ePTFE membranes and concluded that the genotype did not affect the treatment outcome in the first year of follow up (*p*>0.05). However, in the fourth year patients susceptible to periodontitis exhibited significant CAL loss (*p*<0.002), significant PPD increase (*p*<0.001) and more unstable regenerated attachment compared to negative genotype patients.

The patients of Christgau *et al.* (26) and Cortellini and Tonetti (28) were controlled for the same IL-1 genetic polymorphism. Both studies used the same therapeutic intervention of guided tissue regeneration. Non-resorbable or absorbable membranes were used for the intrabony defects’ treatment, while Cortellini and Tonetti (28) combined the absorbable membranes with alloplastic biomaterials. The study of Christgau *et al.* (26) included 18 non-smokers, Caucasians with positive genotype patients. They found no significant association between the genotype and the clinical and radiographic outcome after GTR treatment. In addition, no significant influence of the IL-1 genotype was recorded on the clinical outcome of periodontal regenerative therapy of 86 patients in another study (28). In the particular study 32 (37.2%) of the individuals included were genotype positive and the mean follow up period was 7.5 years for the IL-1 positive subjects and 8.7 for the IL-1 negative.

Allografts, xenografts or alloplasts bone grafts were employed in the treatment of inter-proximal periodontal defects of Caucasians and Hispanics in a study published by Weiss *et al.* (27). 13 of the 44 (29.5%) included individuals were found susceptible to periodontitis after genotyping. The genotype positive patients had a mean age of 47 years and four of them were smokers. This study showed that no association was found between the genotype status and the outcome of the bone grafting. IL-1 positive patients exhibited less PPD reduction and more clinical attachment gain than the IL-1 negative patients, but the difference was not found statistical significant (*p*>0.05).

Bias assessment were assessed by the use of the modified NOS-Scale regarding the study design (selection, comparability, outcome and statistics). Bias assessment revealed that the quality of the included studies ranged and a significant heterogeneity of the quality according to the Newcastle-Ottawa Scale found. The scores of the included studies are presented in  Table 3.

**Table 3:**
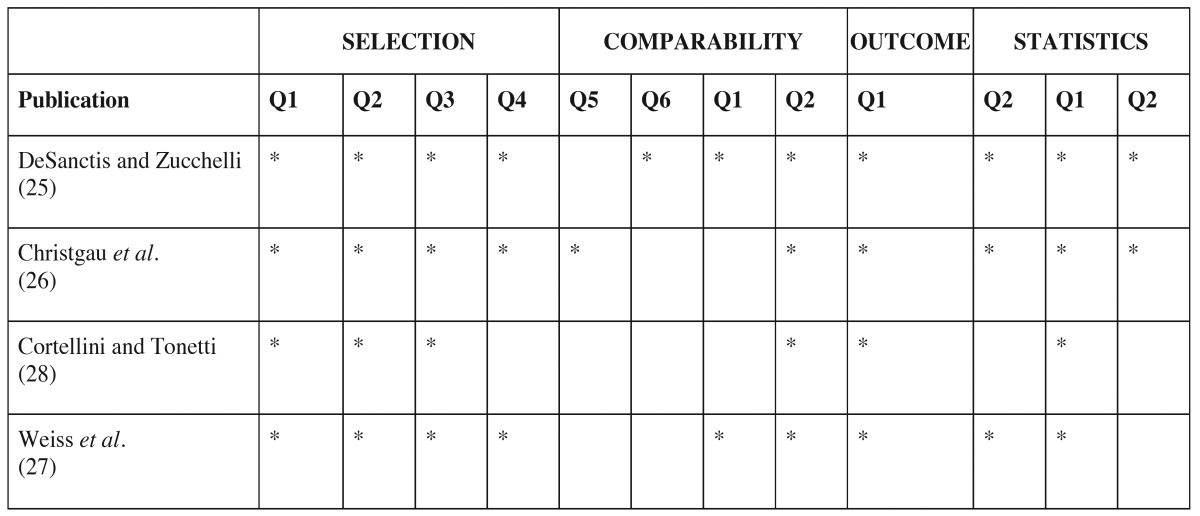
Quality assessment of the included studies.

No meta-analysis of the data from the included studies was performed. Apart from the small number of included studies and the reduced clinical significance of such an effort, several differences in the treatment protocols and the quality assessment were identified between the studies.

 Also presented in  Table 1 the characteristics of the study design for each of the included studies. In one study (27), regenerative procedure consisted of bone replacement grafts, while in the other three included studies guided tissue regeneration with different types of membranes was employed.

## Discussion

- Summary of evidence

No definitive conclusion, based on combination of the of the included studies’ data, can be provided. However, most of the included studies conclude that the clinical outcome of the periodontal regenerative procedures is not influenced by the presence of a susceptible genotype to periodontal disease such as IL-1. On the other hand, one study (25) reported a greater impact of the IL-1 genotype in the long-term stability of the clinical outcome in terms of sustained attachment levels. From those results it can be concluded that it seems that no additional negative effect of IL-1 genotype in regenerative periodontal treatment exists although better controlled studies should be employed. The lack of well controlled studies evaluating the relationship between IL-1, or even other genotypes less popular today, may shed some light in the individuals’ healing variations often observed after regenerating procedures. Although we may stand at an early time point, and this can be reflected in the small number of studies included in the present review, however there is increasing interest for patients exhibiting susceptible genotypes as the regenerative procedures are now employed in the everyday periodontal practice.

Christgau *et al.* (26), Cortellini and Tonetti (28) and Weiss *et al.* (27) concluded that there is no influence of the IL-1 genotype on the clinical outcomes. De Sanctis and Zucchelli (25) recorded an association of the genotype with the clinical result after regenerative treatment. In this study, an influence of the genotype on the stability of the gained attachment level after the regenerative procedure was reported. IL-1 positive patients were more prone in loss of CAL in the regenerated defect between the first and the fourth year of the follow-up period. The PPD increase and the CAL loss in the intrabony defects that were treated with GTR exhibited to be statistical significant (*p*=0.0001) in IL-1 genotype positive patients compared with IL-1 genotype negative subjects. Weiss *et al.* (27) wanted to evaluate the stability of the clinical outcome after four years of follow up just as De Sanctis and Zucchelli (25) did. They conducted an analysis in a subgroup of patients that were re-examined four years after treatment. No significant differences were found as far as the stability was concerned between the IL-1 positive and IL-1 negative subjects.

All four studies reported on systemically healthy patients thus excluding systemic conditions as confounders in the treatment outcome. Many epidemiological studies have correlated periodontal disease with systemic conditions, especially diabetes (29). Diabetes mellitus and periodontitis are interrelated and each one of them affect the clinical outcome of the other (30). Moreover, periodontal disease onset and progression might be influenced by other systemic diseases such as obesity, metabolic syndrome and osteoporosis (31). A potential effect of the aforementioned factors on the clinical outcome of regenerative periodontal therapy, led to the necessity of including only systematically healthy individuals (31).

In the included studies the investigators preceded non-surgical periodontal therapy (scaling and root planing) including oral hygiene instructions before the surgical intervention for ensuring elimination of etiological factors and control of the inflammation. Kaldahl *et al.* (32), showed that scaling and root planing was similarly effective in pocket depth reduction, attachment level change and diminishment of inflammation as surgical treatment in long term follow up (32). Lindhe and Nyman (33) have shown in a longitudinal study that periodontitis can effectively been treated with scaling and root planing and surgical intervention followed by good oral hygiene control in a five-year period (33).

In another study, oral hygiene was found to be a determinant of gingival health maintenance as shown in the study of Cortellini and Tonetti (28) where a 3-month recall program was shown to be effective in maintaining low plaque scores, reduced bleeding in probing, *P. gingivalis* and *P. intermedia* and stable attachment levels four years after guided tissue regeneration in deep infra bony defects. In addition, less stringent supportive periodontal treatment was found to consist a 50 fold up increased risk for attachment level loss (34). The importance of plaque control was also supported by Hellstrom who reported that a stringent oral hygiene regimen with often professional supervision improved the clinical indexes and reduced the subgingival microbiota at infra bony, supra bony defects and furcation sites (35).

Smoking is considered a well evidenced risk factor for periodontal disease, while smoking habits were found to influence the periodontal regenerative therapy as well (36,37). The clinical outcome of GTR and bone replacement grafts was found to be compromised in smokers when compared to non smokers. Based in a subgroup of three studies, a meta-analysis showed statistically significant reduced bone gain (*p* = 0.03) after GTR of intrabony defects in smokers (38). In only one study of the current review (26) the sample consisted of non-smokers, whereas the other three studies (25,27,28) included both smokers and non-smokers. Weiss *et al.* (27) found a non-statistically significant association of smoking and clinical outcome despite the fact that the IL-1 positive patients exhibited less reduction of PPD than IL-1 negatives. De Sanctis and Zucchelli (25) did not perform separate analysis for a possible confounding of smoking habits to the periodontal regenerative result. However the smoker individuals included counted for only eight individuals. It is worth noting that in the study of Cortellini and Tonetti (28) all of the teeth lost in follow up (6) belonged in smokers. Consequently, smokers were found to possess a greater tendency to lose the initial gain when compared to non-smokers. None of the studies however investigated the role of smoking habits for the IL-1 positive and IL-1 negative genotype patients and the clinical outcome of the regenerative therapy.

Other factors that influenced the clinical outcome of regenerative periodontal therapy were reported as well. The baseline PPD of the intrabony defects was found to affect the PPD and CAL after therapy (25), while the full-mouth plaque score was found to influence the gain of attachment levels (25,27). Cortellini and Tonetti (28) concluded apart for smoking, the absence of compliance in periodontal maintenance affects negatively the treatment outcome. Accordingly in particular study (28), five out of the six teeth lost in follow up were attributed patients presenting poor compliance.

As far as the defect morphology is concerned evidence shows that the wall distribution as described by Prichard (39) plays a critical role in the regenerative result. None of the included studies however provided evidence for the characteristics of the infra bony defects either in terms of wall composition or in terms of depth and angle with the neighbouring tooth (40).

Finally, the location of the regenerative site is considered to play a crucial role in the regenerative potential. In the included studies of the current review no information is provided by the authors concerning the location of the sites in the anterior or posterior zone. Only De Sanctis and Zucchelli (25) report on the location of the regenerative site, including 11 incisors 8 cuspids, 6 premolars and 6 molars. In addition, Cortellini and Tonetti (28) report only on the site of the lost teeth, where six out of six teeth lost during follow up were anterior teeth.

- Limitations-Future studies

One of the main limitations of the included studies was reporting on a limited number of patients. The study populations were characterised as small and asymmetric between the IL-1 genotype positive and IL-1 genotype negative patients. In one study (27) only 13 patients with positive genotype were included and the researchers concluded that there was no influence between clinical outcome and the genotype. Consequently, the sample of the studies may require caution in the interpretation of the results.

In the current systematic review, the included cohort studies did not include sufficient number of smokers that could lead to a safe conclusion. The small number of smokers as well as the heterogeneity between smokers as reported by the authors prevented the conclusion on the correlation of genotype and smoking with the clinical outcome of regenerative therapy of periodontal disease.

Furthermore, despite the rapid development of genetics and the discovery of new genes that may be associated with periodontal disease, research evaluating the impact of the genetic factor in periodontal regenerative therapy outcome is lacking. The research in that field is limited to IL-1, while the research around the impact of the polymorphisms in non-surgical periodontal therapy nowadays includes various genes, such as IL-4, IL-6, IL-8, MMP-1, MMP-13 and MBL (41).

Ten years have passed since the last publication that examined the influence of genetic factors on the clinical outcome of periodontal regenerative therapy. In the future, prospective cohort studies with larger sample size should be conducted in order to examine the effects of single nucleotide polymorphisms of various genes on the clinical outcome of regenerative periodontal therapy that is evolving to become an everyday practice of periodontology.

## Conclusions

Within the limits of the present review, no direct conclusion for the effect of a susceptible genotype status (for IL-1) in the clinical outcome after periodontal regeneration could be drawn. However the studies aforementioned provide some evidence that a susceptible IL-1 genotype may not negatively affect the clinical parameters evaluated for a minimum of 6 months following regenerative surgery. The need of more qualitative studies to explore a possible association emerges.
